# Supplementary material for: Loss of all three APP family members during development impairs synaptic function and plasticity, disrupts learning, and causes an autism‐like phenotype
Source: EMBO J. 2021 May 19;40(12):e107471. doi: 10.15252/embj.2020107471 (PMC8204861; doi:10.15252/embj.2020107471)
Supplement: Supplementary file 1 — Appendix [file EMBJ-40-e107471-s003.pdf]

Appendix, Steubler et al. 2021

Table of Contents

Appendix Table S1: Experimental design 2

Appendix Table S1: Experimental design

Test paradigms highlighted with the same color were performed using the same set of animals.

Black color: denotes tests performed with several separate sets of animals.

Order of tests, blue: Patch Clamp, Sholl analysis, Spine analysis.

Order of tests, red: USV, Elevated Plus maze, Home cage analysis (LABORAS). A total of 34 animals underwent testing, however not all of them performed all tests. 29 animals were randomly selected for USV analysis at postnatal day 7 prior to genotyping (15 LM and 14 cTKO). At weaning all animals were genotyped and these 19 LM and 15 cTKO underwent Elevated plus maze (EPM) and LABORAS testing for repetitive behaviors (rearing and climbing). One LM was excluded from EPM analysis due to seizures.

Order of tests, green: open field, grip strength, rotarod, Morris water-maze place navigation, burrowing, nesting, T-maze, radial-arm-maze, Barnes maze, Morris water-maze cue navigation.

T-maze: * 2 cTKOs showed immobility in the starting arm and were excluded from T maze analysis as they performed only very few choices.

Barnes maze and MWM cue navigation: ** one LM female was found dead after 6 weeks of testing and did not undergo testing in the Barnes maze and MWM cue navigation. *** one cTKO was excluded from Barnes maze analysis due to seizures.

	Method	Age [months]	Number of animals (LM)	Number of animals (cTKO)
**Brain anatomy and neuronal morphology**	IHC: layer specific markers	5	4	4
	Stereology (young cohort)	5-6	5	5
	Stereology (old cohort)	18-20	5	6
	Sholl analysis	4	7	6
	Spine density analysis	4	7	6
**Electrophysiology**	Patch-clamp analysis	4	7	6
	Extracellular field recordings	4-5	5	5
**Neuromotor tests and repetitive behavior**	Open Field	5	11	11
	Grip Strength	5	11	11
	Rotarod	5	11	11
	Home cage analysis (LABORAS)	3-4	19	15
**Species-typic innate behavior**	Burrowing	5	11	11
	Nesting	5	11	11
**Memory and Cognition**	MWM place navigation	5	11	11
	T-maze	5	11	9*
	Radial arm maze	5	11	11
	Barnes maze	6	10**	10***
	MWM cue navigation	6	10**	11
**ASD-like behavior**	USV	7 days	15	14
	3 chambers social interaction test	2	26	23
**Anxiety**	Elevated Plus Maze EPM	3	18	15
**Olfaction**	Burried food test	2	15	14
