# Supplementary material for: Loss of all three APP family members during development impairs synaptic function and plasticity, disrupts learning, and causes an autism‐like phenotype
Source: EMBO J. 2021 May 19;40(12):e107471. doi: 10.15252/embj.2020107471 (PMC8204861; doi:10.15252/embj.2020107471)
Supplement: Supplementary file 2 — Expanded View Figures PDF [file EMBJ-40-e107471-s002.pdf]

Expanded View Figures

Figure EV1. APP family, breeding scheme of cTKO mice, and schematic representation of the corpus callosum.

A The mammalian APP family. APP and the two APLPs are type I single-pass transmembrane proteins with a large extracellular domain and a short cytoplasmic tail. APP family proteins show high sequence conservation within extracellular domains E1 (green) and E2 (blue), the acidic domain (AcD), as well as in the intracellular domains (AICD = APP intracellular domain, ALID = APLP intracellular domain). APP and APLP2 both contain a Kunitz-like protease inhibitor domain (KPI), while the short Ox-2 antigen domain is specific for APP. Black arrows indicate cleavage sites for α-, β-, and γ-secretase. Note that the amyloid-β (Aβ) domain (orange) is unique to APP.

B Genotype distribution in 38 litters (337 animals in total) obtained after in vitro fertilization and implantation into wild-type foster mothers. At weaning (3 weeks of age), genotype distribution did not differ significantly from the expected mendelian frequency ($\chi^2(1, N = 337) = 1.02$, $P = 0.3132$, ns) excluding embryonic and early postnatal lethality.

C Representation of the corpus callosum (purple) in consecutive slices along the rostro-caudal axis (image credit: Allen institute). In WT mice, the CC extends approximately from bregma +2.0 to bregma −2.5 corresponding to 11–13 consecutive sections in our study.

 © 2021 The Authors

A

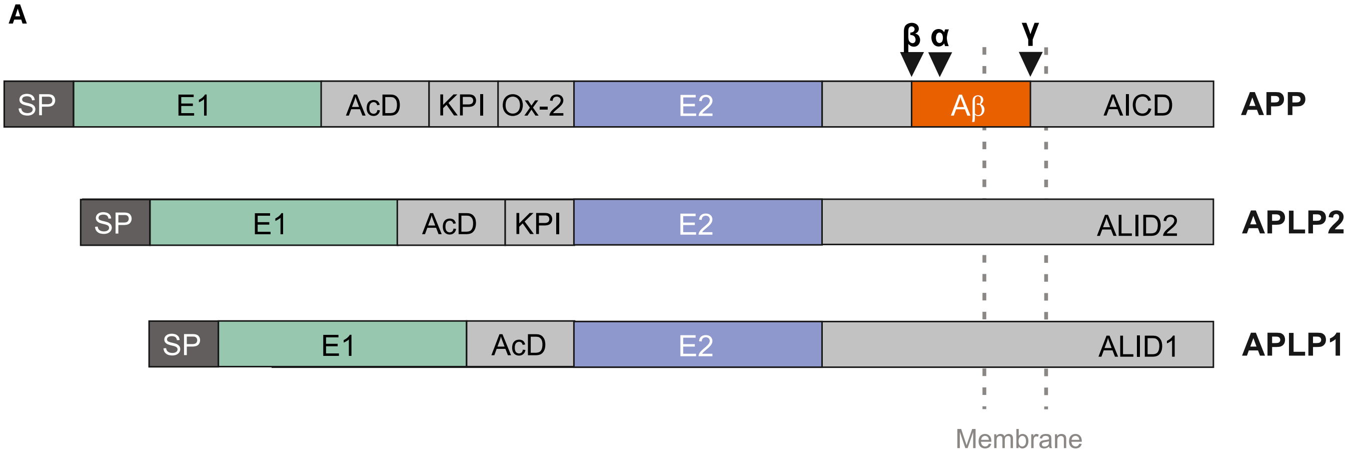

B

Genotype of parents	Genotype of offspring	obtained	χ^2	expected
NexCre$^{+/T}$ APP$^{flox/flox}$/ APLP2$^{flox/flox}$/APLP1$^{-/-}$	„NexCre cTKO“ NexCre$^{+/T}$ APP$^{flox/flox}$/ APLP2$^{flox/flox}$/APLP1$^{-/-}$	156 (47%)	p=0,3132	50%
X				
APP$^{flox/flox}$/APLP2$^{flox/flox}$/APLP1$^{-/-}$	„Littermate control“ APP$^{flox/flox}$/ APLP2$^{flox/flox}$/APLP1$^{-/-}$	181 (53%)		50%

C

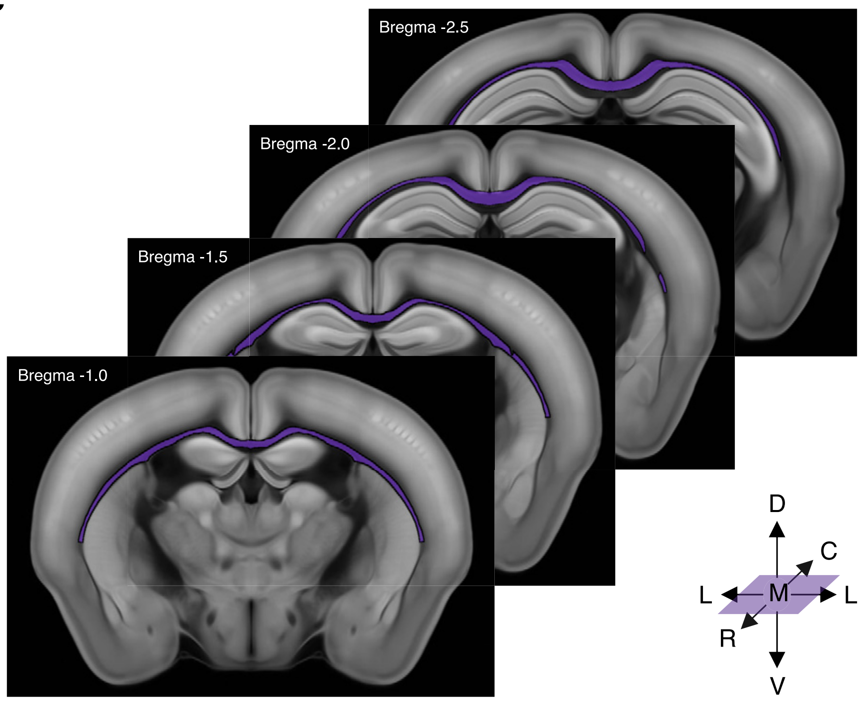

Figure EV1.

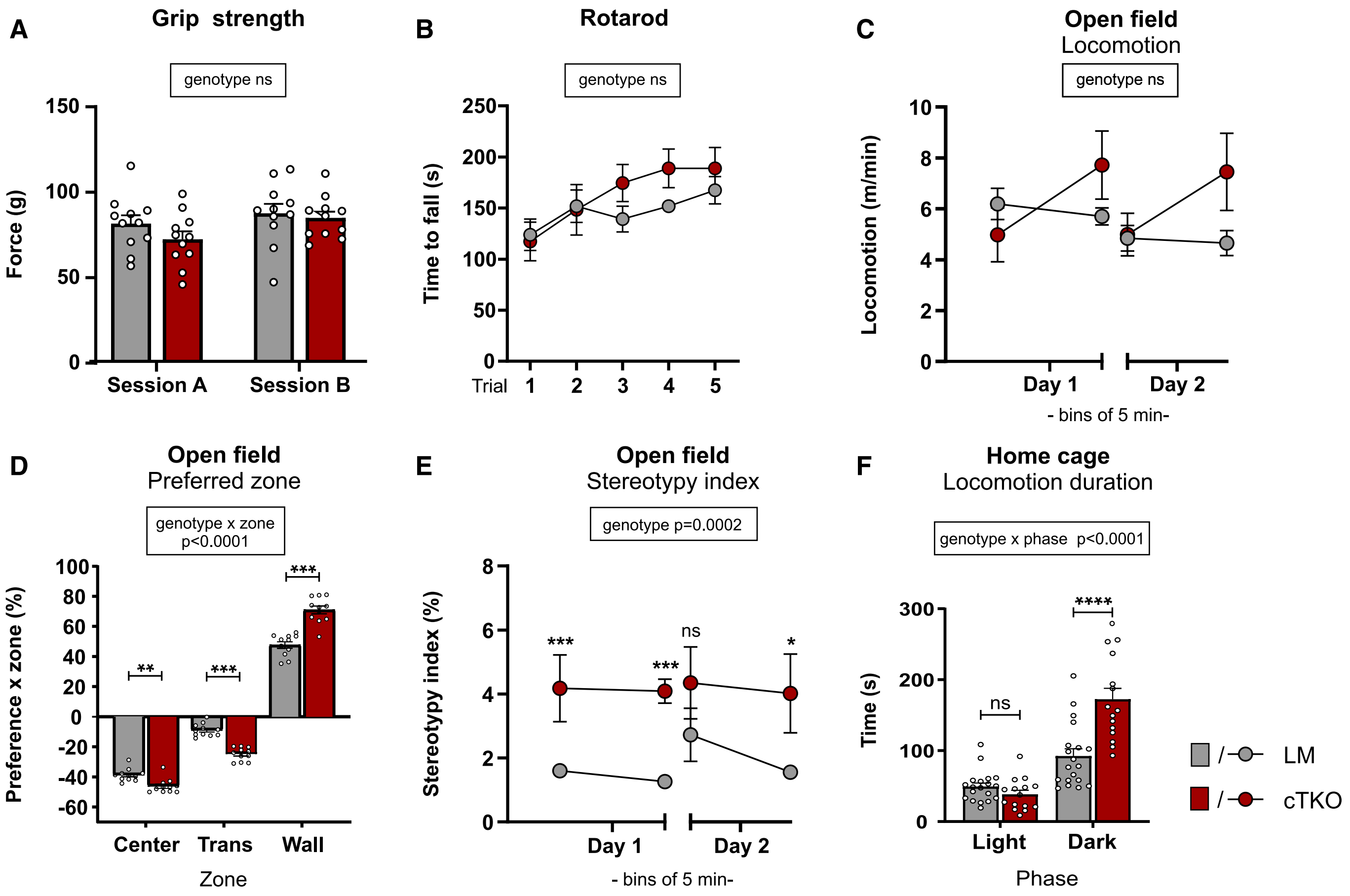

Figure EV2. NexCre cTKOs show normal motor behavior but disturbed open field exploration and hyperactivity in the home cage.

A Grip force of NexCre cTKO animals is undistinguishable from littermate controls in two test sessions [geno $F(1,18) = 1.026$ ns; session $F(1,18) = 9.812$, $P = 0.0058$; session × geno $F(1,18) = 1.208$ ns].

B Rotarod performance is comparable between NexCre cTKO animals and LM controls [geno $F(1,18) = 0.557$ ns; trial $F(4,72) = 11.05$, $P < 0.0001$; trial × geno $F(4,72) = 2.424$, $P = 0.0558$].

C Open field (OF), activity of NexCre cTKOs is similar to controls during exploration of a novel open field arena. Note that NexCre cTKOs did not habituate but showed hyperactivity during the second 5 min on days 1 and 2 [geno $F(1,18) = 0.652$ ns; bin $F(3,54) = 3.982$, $P = 0.0123$; bin × geno $F(3,54) = 5.694$, $P = 0.0018$].

D OF, NexCre cTKOs showed an increased avoidance of the center field and a higher preference for the wall zone [zone $F(2,36) = 1161$, $P < 0.0001$; zone × geno $F(2,36) = 44.13$, $P < 0.0001$].

E OF, assessment of stereotypic behavior. In contrast to littermate controls, NexCre cTKOs show excessive stereotypic locomotion [geno $F(1,18) = 22.76$, $P = 0.0002$; bin $F(3,54) = 0.126$ ns; bin × geno $F(3,54) = 2.623$, $P = 0.0598$. Stereotypy index is expressed as % of the number of 5 × 5 quadratic tiles that were crossed in a repeated sequence over total tile crossings.

F Locomotion in the home cage. NexCre cTKO showed hyperactivity in the dark phase [geno $F(1,32) = 9.329$, $P = 0.0045$; phase $F(1,32) = 126.7$, $P < 0.0001$; phase × geno $F(1,32) = 33.56$, $P < 0.0001$]. $n = 19$ LM animals; sex: nine males, 10 females. $n = 15$ NexCre cTKO animals; sex: five males, 10 females.

Data information: (A-E): $n = 11$ animals/genotype. Sex: six males, five females per genotype. Balanced sex for all groups. Data were analyzed using a mixed ANOVA model and are represented as mean ± SEM. *$P < 0.05$, **$P < 0.01$, ***$P < 0.001$, ****$P < 0.0001$, and ns not significant.

 © 2021 The Authors

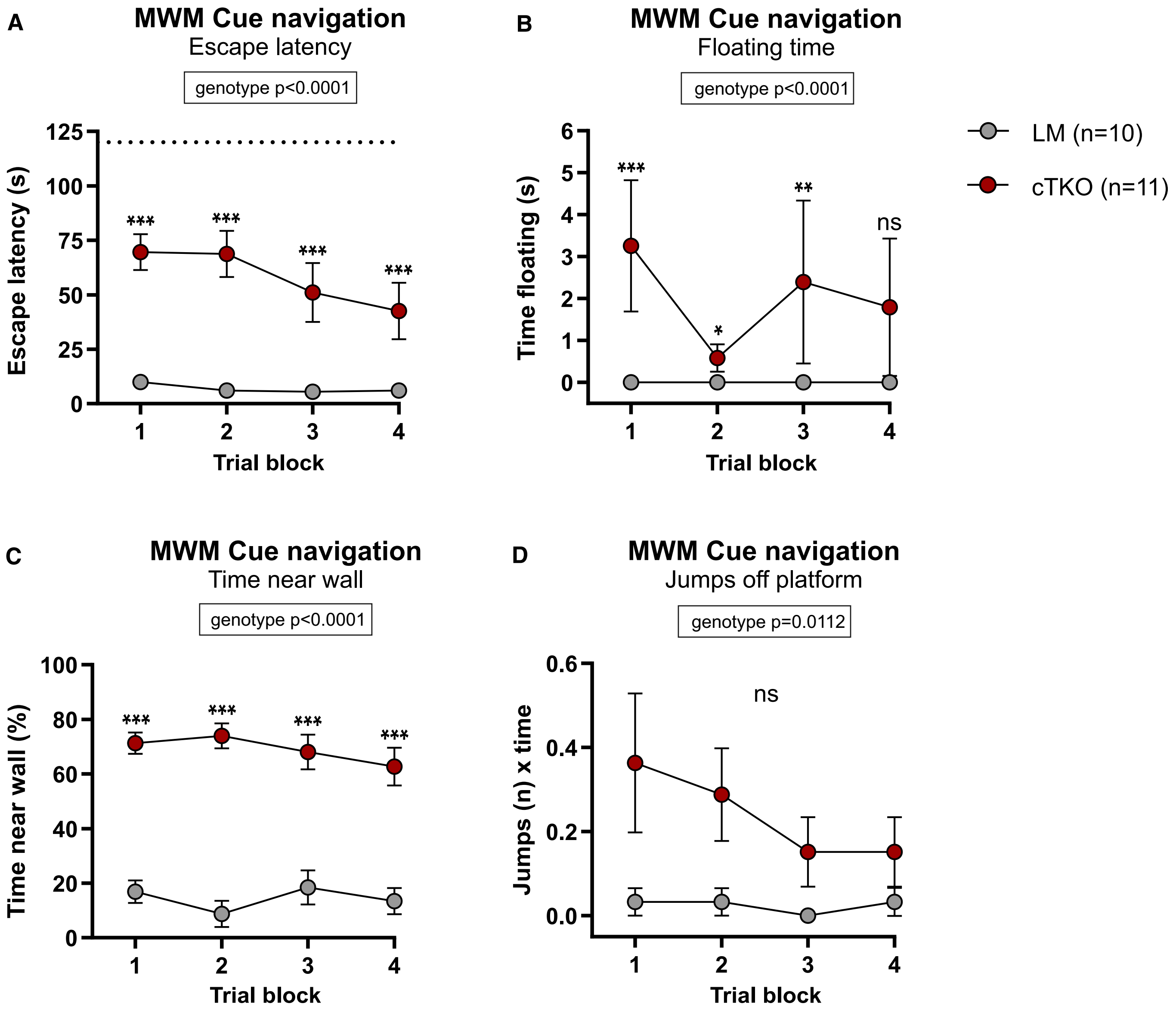

Figure EV3. MWM cue navigation.

A During cue navigation task, NexCre cTKOs show longer escape latency compared to LM controls but do improve during trials [geno $F(1,17) = 124.4$, $P < 0.0001$; trial block $F(3,51) = 9.559$, $P < 0.0001$; trial block × geno $F(3,51) = 2.129$ ns].

B Mutants float more than LM controls during cue navigation [geno $F(1,17) = 33.32$, $P < 0.0001$; trial block $F(3,51) = 11.50$, $P < 0.0001$; trial block × geno $F(3,51) = 10.45$, $P < 0.0001$].

C NexCre cTKO animals spend a high proportion of the time swimming along the wall [geno $F(1,17) = 58.93$, $P < 0.0001$; trial block $F(3,51) = 2.901$, $P = 0.0437$; trial block × geno $F(3,51) = 4.126$, $P = 0.0108$].

D Unlike controls, NexCre cTKO animals frequently jumped off the flagged platform [geno $F(1,17) = 8.097$, $P = 0.0112$; trial block $F(3,51) = 0.784$ ns; trial block × geno $F(3,51) = 0.286$ ns].

Data information: (A-D): $n = 10$ LM animals; sex: six males, four females. $n = 11$ NexCre cTKO animals; sex: six males, five females. Balanced sex for all groups. Data were analyzed using a mixed ANOVA model and are represented as mean ± SEM. *$P < 0.05$, **$P < 0.01$, ***$P < 0.001$, and ns not significant.

© 2021 The Authors

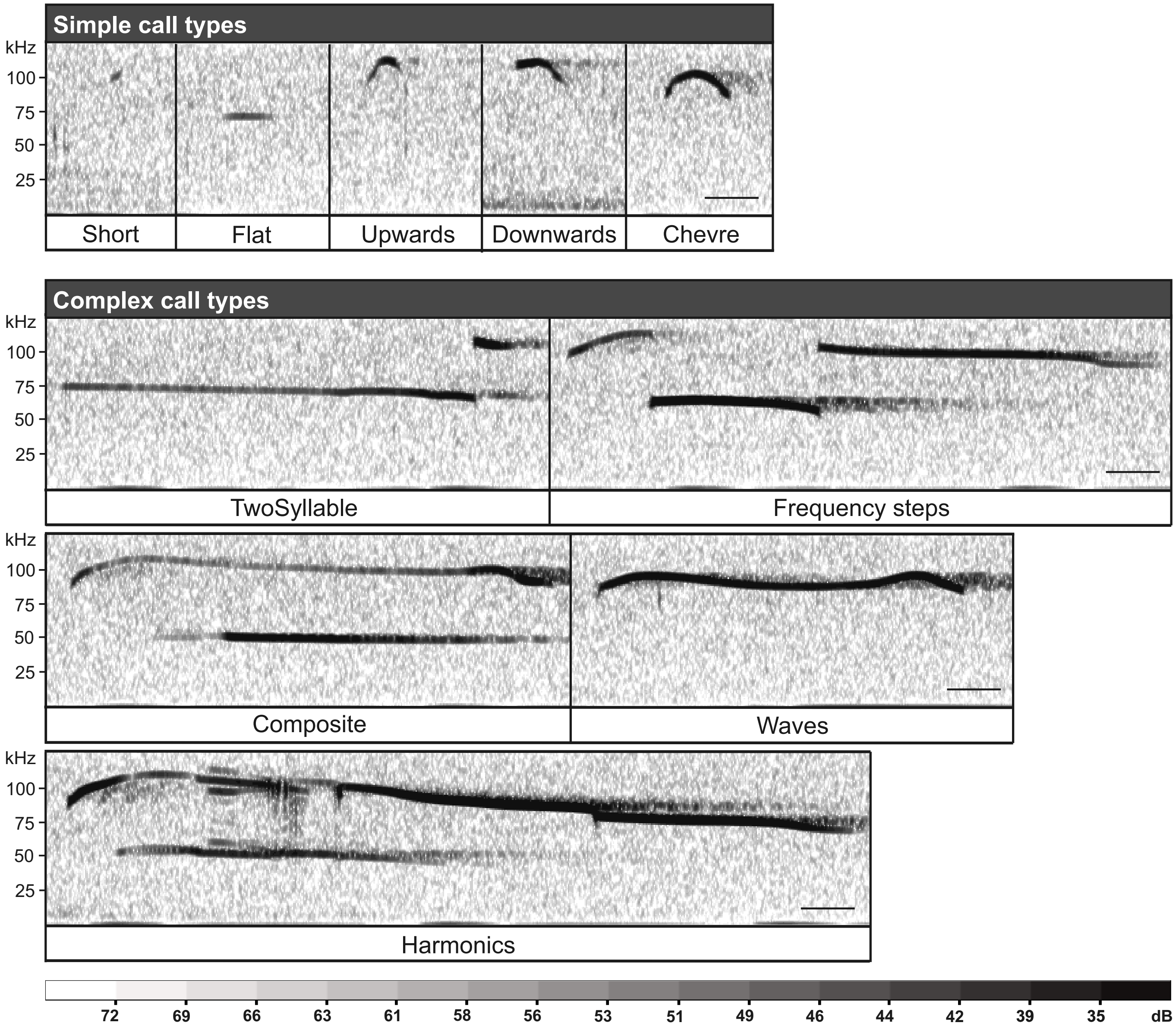

Figure EV4. Typical sonograms of ultrasonic vocalizations and classification of call types.

Representative examples of recorded sonograms of different call types. First row: Simple call types including short (under 5 ms), flat (pitch frequency remains constant), upwards (continuous increase in pitch at least to terminal frequency that is 6.25 kHz higher than start frequency), downwards (continuous decrease in pitch at least to terminal frequency that is 6.25 kHz lower than start frequency), and chevre (initial pitch increase, followed by pitch decrease like an 'inverted-U'). Second-Fourth row: Complex call types, including two syllable (one initial main call followed directly by a punctuated call at the end), frequency steps (several frequency changes without interruption in time), composite (two parallel frequencies), waves (one syllable that consists of two or more directional changes in pitch), and harmonics (a main call that is surrounded from additional calls with different frequencies). Scale bar: 5 ms.

 © 2021 The Authors

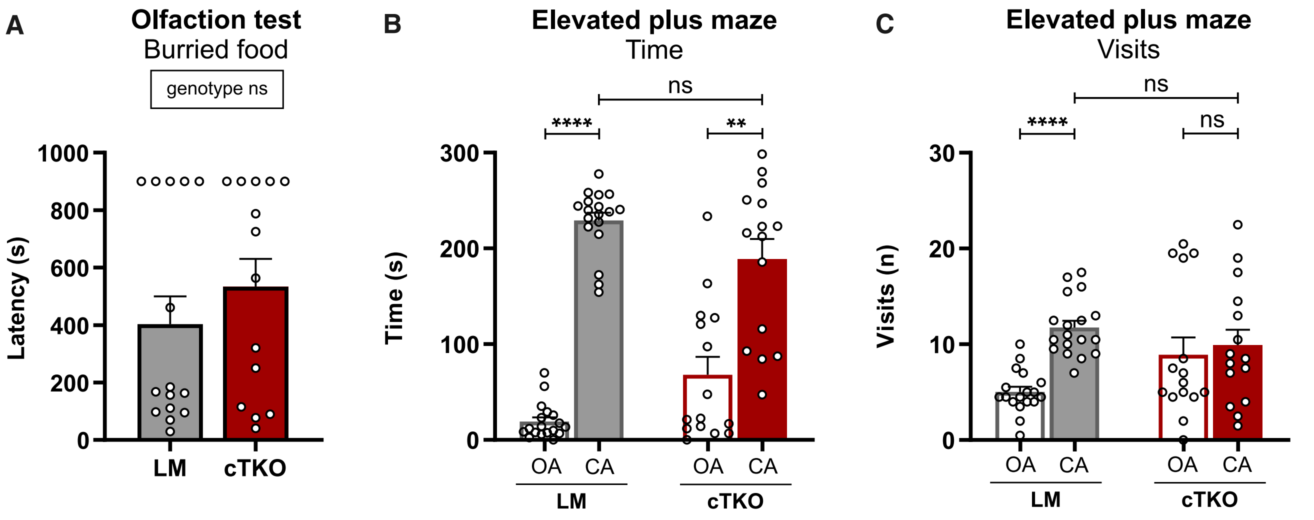

Figure EV5. NexCre cTKOs show normal olfaction and no increase in anxiety.

A Buried food test. Latency to find the hidden food pellet is comparable between LM and NexCre cTKO mice [Mann–Whitney test between n = 15 LM animals, sex: six males, nine females and n = 14 NexCre cTKO animals, sex: seven males, seven females; ns P = 0.5014].

B Elevated plus maze. Like LM controls, NexCre cTKO animals spend more time in the closed arms [paired Student's t-test between open and closed arms of n = 18 LM animals, sex: nine males, nine females, ****P < 0.0001; paired Student's t-test between chambers of n = 15 NexCre cTKO animals, sex: five males, 10 females, **P = 0.0082; Welch's t-test between closed chambers of both genotypes ns P = 0.0903].

C Elevated plus maze. LM visit the closed arms (CA) more frequently, while NexCre cTKOs visit the open arms (OA) and closed arms to the same extent [paired Student's t-test between open and closed arms of n = 18 LM animals, sex: nine males, nine females, ****P < 0.0001; paired Student's t-test between chambers of n = 15 NexCre cTKO animals, sex: five males, 10 females, ns P = 0.5689; Welch's t-test between closed chambers of both genotypes ns P = 0.3095].

Data information: (A-C) Data are represented as mean ± SEM. **P < 0.01, ****P < 0.0001, and ns not significant.

© 2021 The Authors
